# Enacting Media. An Embodied Account of Enculturation Between Neuromediality and New Cognitive Media Theory

**DOI:** 10.3389/fpsyg.2021.635993

**Published:** 2021-05-25

**Authors:** Joerg Fingerhut

**Affiliations:** Berlin School of Mind and Brain, Department of Philosophy, Humboldt-Universität zu Berlin, Berlin, Germany

**Keywords:** 4E cognition, architecture, artifactual habits, digital media, film, neuromediality, picture perception, predictive processing

## Abstract

This paper argues that the still-emerging paradigm of situated cognition requires a more systematic perspective on media to capture the enculturation of the human mind. By virtue of being media, cultural artifacts present central experiential models of the world for our embodied minds to latch onto. The paper identifies references to external media within *embodied, extended, enactive*, and *predictive* approaches to cognition, which remain underdeveloped in terms of the profound impact that media have on our mind. To grasp this impact, I propose an enactive account of media that is based on expansive habits as media-structured, embodied ways of bringing forth meaning and new domains of values. We apply such habits, for instance, when seeing a picture or perceiving a movie. They become established through a process of reciprocal adaptation between media artifacts and organisms and define the range of viable actions within such a media ecology. Within an artifactual habit, we then become attuned to a specific media work (e.g., a TV series, a picture, a text, or even a city) that engages us. Both the plurality of habits and the dynamical adjustments within a habit require a more flexible neural architecture than is addressed by classical cognitive neuroscience. To detail how neural and media processes interlock, I will introduce the concept of *neuromediality* and discuss radical predictive processing accounts that could contribute to the externalization of the mind by treating media themselves as generative models of the world. After a short primer on general media theory, I discuss media examples in three domains: pictures and moving images; digital media; architecture and the built environment. This discussion demonstrates the need for a *new cognitive media theory* based on enactive artifactual habits—one that will help us gain perspective on the continuous re-mediation of our mind.

## Introduction

Media are the core currency of culture. Alongside images, texts, and sounds, new varieties of media (especially in digital form) profoundly shape human “pattern practices” (Roepstorff et al., 2010) across cultural domains. In these contexts, situated cognition is well-placed to examine how sociocultural niches scaffold and structure the mind. Yet paradoxically, media phenomena do not occupy a central place within the discourse on situated cognition. In this paper, I propose an understanding of our engagement with media artifacts based on a theory of habits. To explain such habits, I take my cue from enactivism and recent theories of embodied or radical predictive processing (Clark, 2013, 2015a). I demonstrate how such an understanding is needed to capture the disparate ways media artifacts engage us with their experiential models of the world. I center artifacts as an object of study due to their status as the most quintessential and enduring manifestations of human culture. Exploring a systematic media perspective about such artifacts ought to inform (and form an integral part of) situated cognition accounts of enculturation.

Enculturation is commonly understood as the acquisition of cognitive practices within sociocultural niches, covering ontogenetic levels of dynamic change that unfold across a lifespan. Such ontogenetic niches have been the focus in cognitive science and will be the focus of the present paper as well, as it mostly deals with media in what has been labeled “developmental” or “cognitive niches” (Stotz, 2010; Bertolotti and Magnani, 2017). Given its discussion of cultural evolution and cultural development, theoretical discourse on enculturation constitutes a significant addition to *embodied, embedded, extended*, and *enactive* (4E) cognitive science (Hutchins, 2011). Accounts for enculturation claim that “culturally mediated worlds in which we grow up and live are integral to how our brains achieve their functional capability” (Kirmayer et al., 2020, p. 6). This occurs holistically. For example, “cognitive integration” theories link the acquisition and entrainment of capacities for calculation to wider practices that encompass epistemic tools and representational systems within a culture (Menary, 2007, 2018).

While such accounts are theoretically invaluable, they typically focus on higher-level cognitive capacities (at least when considering paradigm cases). This includes capacities that are only made possible through cultural practices (Hutchins, 2008), as well as specific epistemic operations derived from certain tools and media. Among these are those relating to the capacities of reading, writing, memory, and mathematical cognition (Heyes, 2012; Menary, 2015; Fabry, 2018). Such accounts are not immediately concerned with broader questions regarding cultural tools and media, such as how they might afford novel, experiential models of the world. Moreover, the field does not sufficiently engage with human artifacts and media beyond notational systems and language. Other cultural artifacts, such as images and films, new and digital media, and the built environment, could be considered as equally central and pervasive insofar as they substantively structure our cognitive lives—they even permeate our perception and affectivity. By focusing on how we enact such artifacts, this paper aims beyond a single cognitive practice (made possible by the processes of enculturation) to explore how experiential domains are generated through embodied media habits.

Although the cognitive sciences routinely consult the theoretical traditions of philosophy and psychology, they often overlook relevant theoretical work in fields such as image science and media studies. This is unfortunate because media studies, especially, could be an important humanities companion to 4E cognitive science. As a field, media studies elucidates the inner operations and logics of different media systems. In doing so, it reveals the relevance of media’s technological dimensions to our lives. After all, media are artifacts that expand our cognitive and experiential reach beyond traditional conceptions of the human senses. Media record, process, and transmit information. As media studies have shown, these basic operations developed over history in different cultural-technological niches and became implemented in specific forms. The prominent field of *media archeology*, for example, traces the trajectories of technological devices such as the typewriter, film, and computers (Kittler, 1999). Media theories therefore emphasize the material and technological underpinnings of media (Gane, 2005) while also showing how media amount to more than that. As Kittler asserts, “media determine our situation” (Kittler, 1999, p. xxxix). The guiding premise for the present paper, then, is that both 4E cognition and media studies capture the ways in which cultural artifacts shape our lives and minds.

I argue that the embodied habits and skills employed when engaging cultural artifacts constitute a central level of description (Fingerhut, 2020a). Habits are ways of acting. As such, they structure our perceptions, emotions, and thoughts. Habits are also expansive in three aspects: time, space, and the sphere of activity they afford. (a) Habits assemble tacit expectations within certain ecologies and therefore structure our future actions therein. Since those expectations have been shaped over time, habits link our current engagements also with our history of environmental coupling. In other words, they are *temporally expansive*. (b) Habits are co-constituted by our socio-cultural-technical environment, making them *locationally expansive* in the sense that, for example, media artifacts critically determine the way an engagement unfolds within a habit. (c) Interestingly, habits (which are often seen as exhibiting an inherent inertia) further exhibit a tendency to transcend themselves. They do so by adapting to novel circumstances or by unlocking new domains of interaction. This means they are *transformatively expansive*.

More specifically, this paper will explore the sensorimotor and body-schematic processes underlying artifactual habits with respect to pictures and cinematic productions—along with new (social and digital) media as well as the built environment (the lasting impact of which is re-mediated in our smart cities). Clearly, the processes constituting a habit are more complex and varied than such a focus can reveal. Higher cognitive processes also play a central role in the unfolding of skillful engagement, as is addressed by so-called ‘vertical elements’ in *meshed architecture* accounts of skills (Christensen et al., 2016). Such processes go beyond the scope of the present paper, for its focus is not so much cognitive control but rather to what extent control over experiential engagement—in a more bottom-up fashion—is exerted by the medium and the interaction itself (Gallagher and Varga, 2020). The general emphasis is on medium-specific habits (i.e., how media habits differ from one another and how they are adaptive in specific media ecologies), the ways pervasive artifacts permeate our cognitive engagement down to the level of perception and affect (think of the impact of architecture, film, digital media), and how this pans out in encounters with specific media works (i.e., how we attune to media and how they entrain us in the *here and now*). With this emphasis, we can identify central elements of our engagement with the experiential models presented to us by media.

The first section of this paper briefly surveys some 4E cognition accounts that reference media to provide an understanding of the nature of the mental states that emerge when media engage human organisms. One focus will be the hybrid realization claims of the e*xtended mind*. Another focus will be the *enactive* nature of our mental states and the evaluative domains such an enactivism entails. I will subscribe to an enactive account of habits that highlights the active role of the body in bringing forth experiences with the purpose to extend this idea to media ecologies. Yet I will also discuss how this account can retain a focus on the hybrid material nature of the brain-body-nexus underlying such engagements and its structure (in broadly functionalist terms), which some variants of enactivism might reject.

The second section addresses the role of the brain in our media engagements more directly. Understanding the way our neuronal processes dovetail with media on different levels of the hierarchical processing of the brain (along with how this relates to the way information is recorded, processed, and transmitted in different media) could be taken up by *radical predictive processing* theories (Clark, 2013, 2015a). These theories give an account of the role that so-called designer environments (and media, in my understanding) play in the dynamics between the brain, body, and world. My focus here will be on active inference and design-guided bodily engagement. Within a habit, then, we can identify *neuromedial* elements that complement the unfolding of a media engagement. Such a framework presents itself as a theory of media as central experiential models of the world that need not be mirrored in the brain, but rather engage the brain-body nexus.

Section three recounts this idea and relates the situated mind to a general media theory. All this has implications for how we should conceive of our more specific media engagements. Section four therefore discusses examples of media engagement types (exploring also the mental states realized *within* an artifactual habit) and prepares the grounds for a *new cognitive media theory* based on what has been discussed before. It then associates these types to the medium-specific body schema we employ when engaging with film, to the capacity of *seeing-in* with respect to pictorial artifacts, to the ways new and digital media actively engage and predict their users, and – last but not least – to the understanding of the built environment as a media environment.

## Situating Media in Theories of the Mind

Philosophy of mind is media theory. This is true in a general and rather trivial sense. What reaches our mind is mediated by our body-brain nexus and habits of interacting with the environment that we have acquired over time. Within a relational, situated philosophy of mind, the central function of the brain is one of a “mediating organ” (Fuchs, 2011). This organ facilitates engagements between an agent and the world, with those engagements themselves now gaining center stage for an understanding of the mind. But what seems trivially true does not translate easily into a theory. This is because a media perspective could erroneously suggest that the mind is a receiver that exists outside of the mediating apparatus—a position I argue against. In the following paragraphs, I will not explore the general concept of mediation, though, but rather focus on the role that external media play in situated cognition accounts and that might shed a light on the relational nature of our mind.^1^

### A Mixed-Media, Deterritorialized Cognitive Science

External media have been most prominently referenced in theories of the extended mind (EM). These theories argue that media artifacts, whether a handwritten notebook or an iPhone, could be taken as literal parts of the machinery that realizes mental states (under specific circumstances, such as the reliability, trustworthiness, and accessibility of the external device). Clark and Chalmers’ (1998) perennial thought example describes a notebook taking over the memory function of the brain of an Alzheimer’s patient named Otto, substituting what would otherwise be carried out by neural realizers in healthy individuals. Beliefs therefore supervene upon a hybrid brain-artifact structure in Otto.^2^

In other writings, Clark emphasizes that external structures may not gain a central role in co-constituting cognition if they did not significantly complement what the brain-body nexus can do on its own: “external structures function so as to *complement our individual cognitive profiles* and to diffuse human reason across wider and wider social and physical networks whose collective computations exhibit their own special dynamics and properties [emphasis added]” (Clark, 1997, p. 179, 1998). Sutton (2010), who refers to such accounts as “second wave” EM, spearheads exograms (Donald, 1991) and the idea of exosomatic memory to drive home the point of complementarity. This latter notion is also core to aforementioned ‘‘cognitive integration’’ theories.^3^ Exograms are external media storage devices, such as written books, images, libraries, databases. These devices do not simply mimic neuronal memory processes (engrams). Instead, they exhibit properties that inner processes typically lack: reliance, transmittability, reorganization, and so on. It is therefore the combination of inner and outer formats that was beneficial in such cases and which enables the human mind, as compared to a species exhibiting a more limited range of such combinations, to achieve novel and exciting things.

As the hybrid structures of EM suggest, mixing media might generally be advantageous. Clark (2019) refers to *DeepMind* or Differentiable Neural Computers, which are highly evolved machine learning systems. These successfully perform tasks by employing a so-called read-write unit that enables them to externalize certain processes in a different media format (by writing them out), thus giving them sensorimotor access to stable yet modifiable external storage elements (Clark, 2019, p. 272). This describes a cognitive solution that uses engrams and exograms alike. Such an artificial system might seem a rather alien example (albeit one that gains significance when we think of the effects of AI, ubiquitous computing, and the digitalization of our life world). Yet the example demonstrates how mixed systems, understood as one media system exploiting another, jointly constitute better cognitive solutions.

Sutton suggests also a third wave of EM: for human brain-body-artifact interaction we could consider dynamic “shifting networks of heterogeneous components temporarily clustered or clumped together in contingent coalescence” (Sutton, 2010, p. 194). This has further consequence for how we should study cognition:

If there is to be a distinct third wave of EM, it might be a *deterritorialized cognitive science* which deals with the propagation of deformed and reformatted representations, and which dissolves individuals into peculiar loci of coordination and coalescence among multiple structured media [emphasis added]. (Sutton, 2010, p. 213).

With such a wave, we would study series of transformations occurring in interactions between human organisms and artifacts as temporal integrations. These integrations allow for de- and reformations as part of the cognitive process, before then fading out again.

Given the perspective on media proposed here (as a description of how we attune to media), it is tempting to follow a third wave of EM that highlights fleeting “soft” or “transient assemblies” (Clark, 1997, pp. 42–45, 2016, p. 150). This is because the cognizing organism need not be the center of control nor the sole focus when it comes to the kind of information processing involved. The organism also cannot claim agency in such assemblies (Kirchhoff, 2012). Consider smartphones and the other touchscreen devices that we carry around with us: they entrain us when we watch a video, for example, by providing a filmic exploration within a respective media-specific succession of frames (aided by sound and music). Yet they can also re-direct us to their surfaces, such as when we receive a message. Whether in entrainment or in the switching of attention, the activity is elicited and structured by the multi-media device. Similarly, consider how so-called smart cities of today aim to engage us (often also via screen-based media): they steer our movements and elicit cognitive processes by nudging our behavior and using their own algorithms to engage us (they do so more actively than traditional architecture, which already engages us by guiding our embodied exploration of space).

These examples make obvious that something else might be required beyond transient assemblies. In order to capture more fully the nature of our media engagements, we need to identify the central constraints that determine a specific kind of media engagement. We therefore have to focus on recurring media or artifact coalescences and the ensuing structured interactions they elicit. While it is true that an encompassing theory of enculturation also has to understand what it means for real-time coalitions of organism and artifact to mix and dovetail, it is as central to relate such media-mixing and reformatting of information to the more enduring habits sustained in specific media ecologies. Those habits determine our engagement with pictures, screen-based media, and the built environment, etc. Rather than therefore fully *deterritorializing* cognition (as third wave EM seems to suggest), this rather requires an enhanced focus on the cultural contexts that provide structure along with the recurring ways pervasive artifacts entrain us. What therefore is required is the mapping of multiple (often dormant) skills and habits that are constantly re-activated and re-negotiated upon exposure to a media environment, which I will address below.^4^

None of the above waves of EM amount to a theory of cognition on their own. Instead, they present some arguments that should inoculate us against simply assuming that cognitive processes are confined to the skull and skin and exclusively realized in a specific neural medium. The hybrid realization view of EM has relevance for the present paper as an epistemic claim: by giving up the focus on the locally instantiated brain-body, cognitive science should be rewarded with extra explanatory power and parsimony. We can track how in certain media engagements, organism and artifact jointly explore a content. The brain does not have to mirror the operations of the medium, but simply to latch onto them (as I will explore in section “The Radically Predictive Brain”). With respect to mental states such as beliefs, EM attributes a co-constitutive role to external media. But the hybrid realization view that underlies this move also connects with a broad functionalist commitment in EM (Wheeler, 2012). This commitment is not shared by other Es, as we will see shortly with respect to enactivism. They claim that what is central to cognition is not captured well by a computational description of information-processing (and thoroughly misrepresented when relying on representations).

Another well-known challenge to EM comes from internalists such as Adams and Aizawa (2001). This challenge grants that extra-bodily elements may indeed *cause* cognitive processes, but that we cannot infer constitution from causation. For our media cases, the question is: do operations outside the organism *co-constitute* the exploration and bringing forth of world models (such as sensorimotor loops structured by the filmic medium or those co-processed by artificial computations in virtual reality setting)? Or is it that the brain only *causally depends* on such media-body-brain couplings and rather the more local brain-body nexus realizes cognition? It is worth noting that cognitive media theorists who subscribe to 4E claims highlight the transformation of cognitive capacities through media (such as extended empathy in film; Smith, 2012; see also section “Seeing-in Pictures”) and the centrality of embodied engagement (Nannicelli, 2019). Yet they mostly do so without assuming a literal extension of cognitive processes into the media artifacts we engage with, as EM would have it. According to such theorists, cognitive capacities and affective relations are still realized locally within an embodied agent.

The present paper does not focus on boundary definitions for cognitive systems. Also, specific media may pose additional challenges to an EM account for media interactions.^5^ Yet I will return to some of those issues when discussing artifactual habits that are *locationally expansive.* There, I argue that a parsimonious theoretical assessment of certain media engagements captures organism and media as *jointly* exploring and bringing forth meaning or even models of the world. In any case, under the concept of habit otherwise seemingly disjointed processes (inner-organic and media processes) can be understood as unified (Fingerhut, 2020a). Although I do not focus on ontological claims regarding constitution and causality, I want to at least hint at a (in my view) promising way to challenge previous renderings of constitution. This could be accomplished by including dynamical and reciprocal causality between organisms and environment as part of what counts as constitution (Kirchhoff, 2015).^6^ I generally agree with such accounts, which argue that the diachronic element of our history of engaging with artifacts (captured by the *temporal expansiveness* of habits) also has some bearing on the *locational expansiveness* of a mental state, as I will address below.

### Enactivism and Domains of Value

As I argue in this paper, media engage us in an active exploration. A mainstay of enactivism is that perception and experience should primarily be understood as the activity of an organism. At the core of the enactivist approach (Varela et al., 1991; Thompson, 2007; Di Paolo and Thompson, 2014) lies the idea that we should understand all cognition as meaning-making against the backdrop of self-organized autonomous systems and their structured interactions with the environment they bring forth. Enactivism therefore unfolds around the concept of the metabolic organism and the autonomous self. It claims similar principles hold for single cells in their chemical environments, bodies-plus-tools in more evolved organisms, and human agents in social settings. The autonomous, living body is nonetheless at the heart of such accounts, which are organism-centered and that model cognitive activity in terms of its relevance to the viability of an organism. “Cognition, in its most general form, is sense-making—the adaptive regulation of states and interactions by an agent with respect to the consequences for the agent’s own viability” (Di Paolo and Thompson, 2014, p. 76). Here, cognition is understood as a temporally extended dynamic and as an ongoing adaptive regulation.

The central cognitive activity is sense-making. This activity captures what we do when we bring forth meaning. Within such a concept, environment and organism can be seen as occupying co-constitutive roles (Thompson and Stapleton, 2009). The history of structural coupling between organism and environment leads to a form of convergence between the two, defining also what an organism is sensitive to in its environment.^7^ Adaptivity is therefore also centrally interwoven with the sense-making of an autonomous system, in which it tracks whether environmental conditions are beneficial or detrimental for its viability (Di Paolo, 2005; Di Paolo and Thompson, 2014). The mutual co-determination of organism and environment occurs on evolutionary and ontogenetic timespans. But crucially, it is also is present in the immediate dynamics of the *here and now*. The latter is highlighted, for instance, in theories of “participatory sense-making” (De Jaegher and Di Paolo, 2007). Whereas some earlier accounts of autonomy-based enactivism focused on coupling with the environment mostly from the viewpoint of the organism, participatory sense-making gives socially negotiated cognition center stage—dispensing with the idea that relevant cognitive activity originates solely from a single organism.

Individual and interactive levels here are mutually enabling. Recent enactive accounts of language can be additionally seen as a media related extension of participatory sense-making. These accounts reference a central cultural domain within the human social niche: “linguistic sensitivities are the result of the specific contingencies and ecological co-constitution of our bodily existence in human worlds” (Cuffari et al., 2015, p. 1199). Yet despite this interest in the ecological constitution via “languaging” (Di Paolo et al., 2018), such accounts ignore both the printed word and the use of language in other media systems as a factor in developing those linguistic sensitivities. Media (e.g., film, screen-based digital media, and printed words) entrain us in ways that are quite different compared to those of embodied, social languaging encounters ‘in the wild.’ But both the specific capabilities we develop with respect to these media along with ways language capabilities might transfer across media boundaries and into participatory sense-making constitute central questions for a media-informed enactivism.

This paper emphasizes the generation, sustaining, and active perception of values within an environment in structural coupling, by focusing on such coupling in media environments. Although the living body is a central reference point for enactivism, the enacted environmental loop it undergoes largely determines which mental state we entertain at a certain moment. That is what I will focus on by disclosing the sensorimotor and body-schematic dimensions of enactive sense-making in media contexts. As certain versions of what has been labeled *sensorimotor enactivism* argue, entertaining an auditory experience (to take just one example) differs from a visual experience based on the mastery regarding patterns of regularity between motor acts and sensory feedback (O’Regan and Noë, 2001). Both experiences differ, for example, from a thought in terms of the type of access to the world that they provide (Noë, 2009). From here, it is a small step to argue that media-sources engage us in media-specific loops with their own forms of access (Noë, 2012; Fingerhut, 2014).

Sensorimotor enactivism has been criticized for unnecessarily relying on (inner) knowledge with respect to the mastery of aforementioned regularities (Hutto, 2005). Despite this difference regarding knowledge, autopoietic and sensorimotor enactivism both agree that mental states cannot be fully captured by functional descriptions (of so-called knowledge obtained by the organism, or even in terms of the functional structures determining bodily loops through the environment). Enactivism could therefore be seen as highlighting the dynamic interactions with the environment more directly (Hutto and Myin, 2020). The specific unfoldings of such interactions is a central component of theories of participatory sense-making and has also been captured by the concept of “attunement” in enactive interpretations of skilled performance theories (Gallagher and Varga, 2020).

Enactivism claims additionally that the ability to generate and sustain values in our environment has to be part of a theory of cognition, proper. This also explains why enactivism relates to EM rather critically (Di Paolo, 2009). The functionalist descriptions that EM brings to bear in capturing mental states (e.g., our beliefs as brain-body-artifact hybrids) are based on the wrong model of the mind. It lacks reference to meaning-making—namely, to the body as a self-individuating system interacting with the environment (Di Paolo, 2009; Di Paolo and Thompson, 2014). Those differences can be unpacked in various ways. One main difference is that the continuous dynamic of regulating and adapting the body in sense-making also entails a concept of value and affectivity that other theories lack (Colombetti, 2017). Such values are sustained at different levels. These include the body in self-regulation, the body in sensorimotor coupling, and the body in intersubjective engagement (Thompson and Varela, 2001; Thompson, 2007; Di Paolo et al., 2018).^8^ Cultural artifacts and media latch onto our bodies with respect to all three modes. For instance, clothing and the built environment alter our self-regulatory processes significantly by providing heat and shelter. Pictures and moving images engage us in a sensorimotor coupling that differs from engagement with depicted scenes in the flesh. They thereby enable us to attribute a different system of values to those scenes. Digital media, in turn, constantly alter our social interactions. Generally, by co-constituting domains of interaction, media embody meaning. This is because they have become part and parcel of the strategies by which the human body engages the world.

While functionalist descriptions cannot fully account for the generation and sustaining of values, I would argue *pace* enactivism that when it comes to the tracking of such values, neuronal mechanisms and bodily sensitivities that enable such tracking constitute a central level of description.^9^ Cognitive neuroscience might therefore capture how our visual system interlocks in perceptual engagements with certain artifacts (how, e.g., a film entrains us). Theories of emotions, in particular, might explain how specific emotional states track values in our environment based on embodied profiles that afford specific kinds of cognitive processing (Prinz, 2004; Fingerhut and Prinz, 2020, forthcoming). When it comes to cultural domains and media, one should think, moreover, of regulatory principles and norms for our bodies to sustain that go beyond avoiding harm and satisfying the need for food or shelter. Our bodies, for instance, might be seen as exhibiting a need for information and exploration. This is exemplified in the affective states of interest and curiosity, which might explain the pleasure we take in a wide variety of domains including media (Biederman and Vessel, 2006). We might therefore also think of further affective and aesthetic engagements that media afford, such as wonder and play, through which we track what we value in the arts (Fingerhut and Prinz, 2018).

### Artifactual Habits

Enactivism argues that we bring forth experiences by engaging with the world and others. Such active engagements differ substantially when we engage with a social scene in a film or explore the world as it is depicted in a photograph. Different pervasive artifacts and media contexts might also have led to the emergence of different bodies (or body-schematic processes) that we bring to bear in such media ecologies. Walking through the built environment of a city, for instance, requires a set of bodily engagements different from the one we employ when seeing a movie in a cinema setting.

By directing attention toward what can be called ‘artifactual habits of exploration,’ I aim to capture the salient differences between those situations. This paper argues that human cognizers are constituted by a plurality of habits that bring forth their own domains of interactions and respective ranges of viability. Habits are structured ways of acting and central loci of meaning-making. It is only when something has either entered into a pattern or is registered as a violation of such a pattern that it becomes meaningful to an organism. The rest is noise. As pragmatist philosophy in particular has acknowledged, habits can therefore be seen as the basic building blocks of the mind: “the medium of habit filters all the material that reaches our perception and thought” (Dewey, 1983, p. 26).^10^

For the present paper, it is central that certain habits can be described as mixed media affairs between bodies and artifacts. This links them to the debate regarding the extended mind and they therefore can be captured by one meaning of *expansiveness* identified earlier. The bodily interaction that pertains to a habit is co-determined by the media artifact. In other words, the engagement unfolds according to media-specific processes. The habit is then re-instantiated each time the brain-body-media coalition is formed. Habits are *locationally expansive* in this sense and in their reliance on external structures of the designed environment, of cultural artifacts, and of media more generally.

Habits also share the quality of being *temporally expansive.* This means they bring our history of environmental coupling to the *here and now*. They thus structure our actions and determine our tacit expectations with respect to a domain (Fingerhut, 2020a). In many ways, habits are comparable to skills. For the purposes of this paper, habits and skills largely function as interchangeable concepts. But in contrast to skills (Fridland, 2017; Hipólito et al., 2020), habits do not require the same level of control in their development. Moreover, they can be acquired and molded simply through exposure and implicit statistical learning. The temporal expansiveness of habits nonetheless exceeds any concept of repetition: “rather than being the repetition of action, habit is characterized as the open and adaptive way in which the body learns to cope with familiar situations” (Miyahara et al., 2020, p. 125).

Habits are not merely rigid mechanical routines. Rather, they constitute flexible ways of world-making and capture how human cognition may be cultural *tout court*: cultural contexts, artifacts, and media latch on to existing modes of perceiving and affective engagement, moving them toward new forms. As such, artifactual habits constitute an interactive domain between organism and environment. Given this, they are determined as much by external media as they are by the activities of the organism. This relates to the third aspect of expansiveness. Artifactual or media habits are proven to be *transformatively expansive*: they generate new patterns of interactions and domains of value in the process of reciprocal adaptation between organism and cultural environment. Some propensity to pick up and integrate new patterns must obtain on the side of the organism (i.e., as an enabling condition), yet artifacts, media, social environments play the more active role in driving such transformations. Technical innovations force us to learn new skills; statistical immersion within new (typically urban) environments or new social media may alter our habits of interpersonal engagement; and finally, cultural innovations and especially the arts may challenge our habits of engagement in various respects.

The account of habits proposed here portrays us as expert performers in different media settings. Synthetic accounts of skilled performance have already addressed some of the competences this entails, along with the flexibility of habits I envision, for other domains (Christensen et al., 2016). For example, Gallagher and Varga (2020) describe a horizontal axis involved in the joint performance of music. This axis stands in opposition to a vertical one involving higher cognitive processes interacting with bodily engagements. The horizontal axis includes processes that “extend into the world, meshed with the structures of our intercorporal and material engagements” (Gallagher and Varga, 2020, p. 7). This is *locational expansiveness*, to use my term. Understanding such attunements and the dynamic, situated processes in performance studies (but also in media context in which we turn out to be expert performers with respect to media artifacts) could centrally inform our understanding of situated cognition as those authors argue.

I discuss examples of media engagements more extensively below, when I put the account of artifactual habits to work (see section “Toward a New Cognitive Media Theory”). But to get an idea, consider cinema. Edited Hollywood movies rely on us exploring their content according to medium-specific patterns. Some of these include specific camera and lens movements or editing techniques that could involve switching perspectives to portray a scene, or a montage to exemplify an idea. Movies are designed by employing film techniques that have evolved over time. Some of these techniques instill immersion in us viewers, which seems to be a central aim of Hollywood cinema, and engage us with configurations that entrain us with their content (a situation, a scene) in specific sensorimotor or affective ways. Despite feeling immersed in such situations it should be clear that these engagements differ significantly from how we could experience a situation or scene in the flesh. We might not be aware of this anymore, but film is contingent upon on us having integrated certain techniques of exploration into our habits of seeing.

With respect to film (as opposed to static images or written text), it is interesting how some of the activity of exploration sides with the medium itself. Film theorists have aimed to capture the ways we lend our body to the medium in such cases. In the process of doing so, it has been argued that we engage a “surrogate body” (Voss, 2011). One way to capture the embodied engagement in these cases is by exploring a specific “filmic body schema” that extends into the filmic realm and expresses itself by engendering certain film-specific embodied engagements (Fingerhut and Heimann, 2017). Some initial thoughts might help demonstrate the plausibility of such a concept. In film viewing, our self-initiated, real-world related movements are attenuated in ways that free up resources for an intensified engagement with the cinematic works themselves experienced as bodily engagement (i.e., with camera movements, editing, perspectival change, as I will address in more detail below, section “The Filmic Body Schema”).

## The Role of the Brain in the Media Mix

### Neuromediality and Media Affordances

Above, I alluded to radical predictive processing (RPP). This perspective weaves “designer environments” into a novel way to understand the brain (Clark, 2015a, 2016). Predictive processing theories generally agree that the central function of the brain is to adjust the organism to its environment by using multileveled probabilistic predictions. In RPP such inner models are seen as action-oriented through and through. They have the function to enable an efficient, and highly context-sensitive grip on structures and scaffoldings in the environment by making “use of multiple, fast, efficient, environmentally-exploitative, routes to action, and response” (Clark, 2015a, p. 18).

In media ecologies, this grip takes on a specific, even more interlocked nature, because media, among other things, have been designed to engage and entrain us. Before going into some of the details of such media engagements, it might be helpful to account more generally for the contribution of the brain in embodied media interactions by introducing the concept of *neuromediality*. Such a concept aims to relate neural activity to artifactual habits of perceiving. By highlighting processes that correspond directly to media engagements, we can avoid falling into a bio-, or socio-essentialism. Such essentialism treats media as something that only impinges on a cognitive system, which itself has evolved and developed in our every-day interactions (e.g., either face to face with others or in the exposure to natural objects) and interprets neural data in this way. Under the proposed neuromedial perspective, neural responses can also be seen as being exapted for media contexts. One aim is therefore to identify neuronal contributions to new dimensions of interaction that cultural artifacts, such as pictures and moving images, afford. The pervasiveness of such media can be speculatively related to the impact of other human artifacts on the brain, which has been explored with respect to the organizational principle of “neural reuse” that has been mostly explicated in relation to tool use and language processing (Anderson, 2010; D’Errico and Colagè, 2018). To date, there are no comparably sophisticated accounts for artifacts beyond language (such as depictions, which arguably occupy a longstanding and central role in human cultures, Brumm et al., 2021).

Notwithstanding such accounts of how neural circuitry integrates new functions, it is generally important for a cognitive science of media to build upon some normal conditions of media-engagement that have developed ontogenetically through experience-based learning and statistical immersion. This is true not simply with respect to images, but also for film and TV, the built environment, and digital media. Such considerations will be instrumental in developing a theory of how artifactual habits differ from each other and how an artifactual habit finds expression in a specific media ecology or cultural environment. They can also help map combinations of media components and the neural-bodily resources on the organism side that they draw on. In a second step, this approach can then address the question of how the quality and content of an experience is determined by habitual patterns of engagement (and the deviations from the norms those habits track)—and how we enact a specific picture, film, or novel.

What do we actually perceive when we engage with media? As I argue, media provide models of the world that a cognizer can latch onto in media-specific ways. Artifactual habits describe such ways of enacting models. Yet it is not the model itself that shows up in our consciousness. Instead, we perceive certain scenes in the forms that pertain to different media (e.g., in pictures, films, and novels), we engage with utterances of other people (e.g., in social media), or we perceive opportunities to move (e.g., in the built environment).

This relates to an understanding of our perceptual system as geared to pick up opportunities to act, which is explored in ecological psychology (see footnote 7). Concepts such as “affordances 2.0” neatly capture how those opportunities to act change dynamically in human-environment systems (Chemero, 2009, pp. 150–4). Here, environmental affordances for action are not just properties available for pick-up to a pre-existing body with specific sense organs (Gibson, 1979). Instead, cultural niche and sensorimotor capabilities are constantly altered on short timescales by human animals acting in these niches. It is in this dynamic sense that affordances have also become a central concept within recent theorizing about the cultural environment and the enticements it contains (Withagen et al., 2012; Rietveld and Kiverstein, 2014).^11^

With respect to different media, then, one could argue that affordances correspond to habits or skills that are the topic of this paper. These central, media-related affordances have to be theoretically modeled in terms of the media-related habits that correspond to them. For example, a depicted door is perceived as walk-through-able in a way that is different from a door in a building. Insofar as media expand our sensory system and co-structure our habits of perception, they also generate new affordances. This pertains to how affordances differ systematically *across* media habits (e.g., the differences between watching a movie, reading a text, or engaging in a social media chat). Another question is how affordances are dynamically modulated *within* a media engagement. The concept of ‘interaction-dominant dynamics’ describes one such dynamic between media artifacts and the brain-body nexus—one that captures how an explorative activity is guided by a media ecology. It has been argued, for instance, that the mouse-computer system entrains the user into a certain pattern of action (Dotov et al., 2010, p. 3). In such cases, neural activity is modulated by the sensorimotor-artifact dynamics of the larger system. This includes switches in processing that could enable the peripersonal space (Làdavas, 2002) of the engaging organism to extend into the virtual environment of the computer screen (Bassolino et al., 2010). After such a switch, the receptive field of certain neurons changes significantly. Objects within the virtual space take on a different presence and the organism engages in a different cognitive processing style. Such kinds of entrainments might even be more intense in new media devices such as virtual reality (VR), where they are used for motor-cognitive neurorehabilitation (Perez-Marcos et al., 2018), yet they can be traced for other media as well.

The point I want to make is of a general nature: understanding different media requires a focus on how media structure our engagement with the worlds we are presented with. We need a view of the brain as sustaining a dynamic and flexible neuro-cognitive architecture (i.e., one that switches between and locks into different media). Here, as before, I suggest the utility of the concept of neuromedial processes for denoting the contribution of the brain in such dynamics without giving it exclusive importance in defining the structure of the relationship to mediated worlds. The way certain media store, process, and transmit information makes them specific model-environments that pre-structure such relations for the human organism. It is—or should be—the task of an enactive theory of media to highlight how we attune to such models and what we can do within them.

### The Radically Predictive Brain

I suggest capturing the dynamic and flexible cognitive architecture in media engagements by philosophical predictive modeling accounts. Clark’s action-oriented version, labeled radical predictive processing (RPP), focuses on the role of the brain in recruiting resources for action (Clark, 2013, 2015a,b, 2016). He provides a theory of the neural system as engaging in active self-organizing dynamics that also could make salient how the active body becomes recruited by designer environments that themselves constitute central models for our mind.

The general idea of predictive coding (PC) is that in terms of perception, cognition, and action, the computational contribution of the brain involves providing a multilayered system that produces predictions or hypotheses about the world. The brain reduces uncertainty about its environment by engaging in “prediction error minimization” (Friston and Kiebel, 2009; Friston, 2010; Friston et al., 2010). The theory assumes that predictions cascade in top-down flows, from higher layers toward lower ones. They are met by upcoming flows of information that either match those predictions or not. The brain deals with incoming information in a cost-efficient way by propagating residual prediction errors in the system (rather than construing a representation based on sensory input).

Predictive coding theories assume that the brain became wired to run an inherently culture-dependent model of the world that controls the body in cultural ecologies through predictive processes (Gendron et al., 2020). Enactivists criticize such predictive theories for their reliance on inner models or ‘priors’ as hypotheses. For them, these bear too much resemblance to inner representations as central explanatory elements (Hutto, 2018; Hutto et al., 2020). Clark (2015a) sees his radical version as being fit to oppose such a criticism, because it treats the brain as mainly engaging dynamical loops through the environment (with the external designer environments constraining these loops, more on this in a bit). He claims that RPP further alleviates explanatory weight from inner generative models (that remain a central element in his theory) by spreading this weight onto the ongoing interactions and the environmental structures themselves.^12^ Along those lines it has been emphasized that one way to reduce prediction error is to test the environment by actively engaging with it, which falls under the concept of ‘active inference.’ Here, the motor system can be described as part of cognition in oculomotor control (for example) as well as in cued and goal-directed movements (Friston et al., 2010; Adams et al., 2013; Constant et al., 2020a).

Clark’s (2013) concept of designer environments directly focuses on how material culture structures our intersubjective take on the world. Public symbols are effectively forcing upon us new regimes of pre-structured, re-entrant information processing.

The same potent processing regimes, now targeting these brand new types of statistically pregnant designer inputs, are then enabled to discover and refine new generative models, latching onto (and at times actively creating) ever more abstract structure in the world. Action and perception thus work together to reduce prediction error against the more slowly evolving backdrop of a culturally distributed process that spawns a succession of designer environments. (Clark, 2013, p. 195).

Clark mostly discusses lingua-form perceptuals that are public, external models of the world (such as language, formula, theories; Lupyan and Clark, 2015). Still, such a view can include media, cultural artifacts, and the larger cultural environment to support claims regarding artifact engagements (Constant et al., 2020a, b). In the quoted passage, Clark’s focus is on cognition and thought. By emphasizing how the structured environment contributes to cognition, he aims to appease the worry that predictive processing does not provide enough internal structure to explain our full-blown cognitive architecture. Yet, what he claims for the “abstract structures in the world” I would argue also applies to the experimental regimes that media present to us. Clark even references different media and their material properties that limit our interaction space (e.g., computer-keyboard interfaces and specific video formats) that are nonetheless key to or cultural ecosystems (Clark, 2016, p. 279–281). In this, they are a central part of the ever-faster succession of designer environments. Media entrain our perception-action cycles. Despite and precisely because they thereby reduce the complexity of (embodied) interactions with our surroundings they also enable us to engage in new and potentially exciting explorations (as we will see with respect to media works such as texts, films, etc.).

The PC framework sees perception as largely operating based on generative models (conditioned probabilities that link data to their hidden causes in the environment) in a top-down way. These operations start with the inward layers of a hierarchical model of the brain. RPP shares this basic assumption, but it enables us to include cultural environments as part of the predictions more systematically. The way the brain dovetails with designer environments could render these environments an outer layer of predictions themselves, generating their own media-specific flow of information. One might still worry that a separation of inner and outer processing is re-introduced, rendering the environments as passive contributors to the inner complex and active machinery. In this scenario, they would function simply as input to the cognitive system.^13^ Another worry is that the ‘free-energy minimization’ that is part of the larger theory unifying biology and cognitive science introduces an overgeneralization that contains the assumption that a system should seek out states and therefore environments that would contain no surprise (known as the “dark room problem,” Friston et al., 2012). Media environments seem to present the opposite of this. Although I do not believe that RPP can fully deal with those worries on its own (for this the larger, more enactive picture form above would be needed), I nonetheless will address some answers from within the framework, because this also helps to see more clearly how media environments could fit into the predictive picture.

#### Active Media Inference

The first worry is that designer environments still seem separated from making a central contribution to cognition. Neural processing of generative models in hierarchical layers of the brain supposedly does most of the work. This worry can be partially assuaged by pointing to the role of action within the active inference concept in RPP and the targeting of different layers of generative models. As we have seen, a central way to reduce uncertainty is to act upon the environment. This allows for an enhanced hypothesis testing. Such a picture is alluring because it can also capture the ways our actions in active inference are pre-structured and limited in designer environments (and media ecologies). It simultaneously addresses how the dovetailing of brain-organism-artifact via this pre-structuring facilitates the organism in engaging with the richness and potency of ecological information.^14^

Once again, consider our brain at the movies and the case of perception. Here, the visual system’s priors are not neutral between many possibilities to engage. Rather, they operate within a limited range of possibilities. In typical Hollywood cinema, for example, we do not have to explore the scene presented on our own: the director, camerawoman, and editor all direct our attention to the salient part of the action. Our eye- and head movements are thus cued (Loschky et al., 2015). In such cases, activity independent of such cues (e.g., saccades to different areas of the screen) would not be rewarded with the relevant information that drives the story. Certain actions, such as standing up and moving toward the screen, won’t yield relevant visual feedback. Seeing to people engage in a movie scene can thus be contrasted with perceiving a scene wherein two people engage in the flesh. Once we have switched to the regime of film (i.e., reduced uncertainty with respect to the more global environment; enabling a specific set of generative models and hyperpriors), we allocate resources to other elements we would not necessarily focus on in real life (e.g., by enhancing our emotional engagement in the close-up of a face). In this scenario, active inference based on sensorimotor filmic priors allow us to engage with an idea, character, and story in ways that would not be available in the real world, especially because certain actions within such a media ecology are reduced and others are taken over by the medium (e.g., by zooming into a scene). Film therefore constitutes its own generative (cause-effect) model. Here, the presence of a medium that adheres to certain regularities in conjunction with layers of neurons engaged in the minimization of prediction error jointly manage the kind of sensory flow within a media habit.

The degree of alignment with an environment that I just described is, for example, captured by variations of “precision weighing” that modulate the impact of error signals in specific contexts (Clark, 2016, pp. 57–59). Precision weighing provides a mechanism that plays a role in what we pay attention to Feldman and Friston (2010); Parr and Friston (2017)—one that has been employed to understanding “presence” in both media and non-media contexts (Parola et al., 2016; Seth, 2019). Take another example. Walking through a built environment (such as an apartment, university, or city) renders certain kinds of information more or less salient. This leads to greater precision, and therefore less uncertainty, in embodied predictions about certain elements. This is, for instance, expressed in a high conditioned probability the streets in a city follow a grid-like structure. Violations within such a geared prediction regime will gain our attention more easily. RPP therefore provides an organism-artifact mixed-media model that, in the end, could be part of an explanation about why certain forms of attention or affective engagement, etc. occur within a specific habit but can be quite different in another media environment. Moreover, the structure of the designed media environments co-constitutes our engagements with generative models in the brain being geared to pick up and integrate recurring patterns.

#### Culture as the Plurality of Mutual Models

Designer environments are thus centrally involved in eliciting switches between generative models in the brain (or what could be considered hyperpriors, such as when switching between perceiving a picture and a social scene *in the flesh*). Even more centrally, however, the external models co-determine the ways in which multilevel, probabilistic models unfold deeply within the engine of the human cognitive system. This view of the cognitive system can therefore do without assuming that we have to represent the structures of the media artifacts themselves. Instead, the brain-body nexus jointly with the medium engages in exploration. The first worry, that of a secondary contribution of media-designs, is thus addressed to some extent. Still, the second worry remains, namely that our engagement with “statistically pregnant” designer environments does not seem to fit the general aim of organisms to reduce uncertainty.

Regarding this second worry, I would like to steer clear from discussions of a dark room that immediately presents itself as an adaptively unreasonable and unsuccessful coping strategy that leaves seekers of dark rooms at an evolutionary disadvantage (it remains problematic that the theory might proposition such a scenario). When it comes to artifacts and media, the more relevant discussion is the perceived value of experiential surprise (Van de Cruys and Wagemans, 2011; Seth, 2019). Predictive Theories based on free-energy minimization do not seem to account for the “deep, positive attractions of novelty, play, and exploration” (Clark, 2018, p. 524). Clark discusses this in terms of an ‘‘information theoretic subversion,’’ which is the idea that we could describe a predictive system maximizing prediction success (avoiding the dark room) and still end up with a perfectly trivial sense in which the system achieves that. Such subversions seem to be forestalled by the plurality and dynamics of our cultural practices, artifacts, and media.^15^ They come to us with new affordances for engagement, with a multitude of complex traditions ready for exploration, and by implicating novel epistemic actions.^16^ Such designer environments thereby ensure “a steady diet of change, innovation, and challenge” (Clark, 2018, p. 531).

This speaks directly to the aforementioned paradoxical aspect of habits as sustaining certain ways of acting while, at the same time, evolving to incorporate new forms of engagement (being *transformatively expansive*). Habits seem to minimize novelty by attuning us to a specific designer environments or media settings. They are therefore conservative in the sense of providing and keeping us within a range of viable actions. Yet since habits are partially constituted by the pervasive artifacts that evolve around us (they are *locationally expansive* in that media co-constitute their exploration), they also can appear as more progressive.^17^ We are exposed to a plurality of designer environments that we co-construe and that still dynamically evolve. In engaging those environments, our inner models and the outer models coalesce. What is more, they become mutual models that span brain, body, and environment, that are actively embodied, and which are shared with others.

These are only cursory remarks. Still, RPP provides an initial theory of how the brain folds media environments into our expansive sense-making activities (with the caveat that it still relies on inner models in ways enactive theorizing would object to, see footnote 12). It claims that the brain-body system picks and engages strategies for dealing with the world based on error minimization and active inference. Media environments, in turn, provide a plurality of strategies for dealing with the world via experiential models, models that constitute the shared space of culture and innovation.

## A Short Primer on Media Theory: The Medium Is the Message

The current paper proposes understanding enculturation by employing a theory about our embodied habits in relation to external media. Here, habits are media-inclusive, temporally outreaching, and governors of the dynamics of our engagement. The premise is that media widen our senses and are central conveyors of culture. Before I discuss how this account of artifactual habits helps us tackle specific media engagements (see section ‘‘Toward a New Cognitive Media Theory’’), it is worth taking a quick detour to see whether the central tenets of situated cognition relate to a more general media theory.^18^

A seminal position within the admittingly diverse field of media theory is McLuhan’s media ecology (McLuhan, 1962) that still promises to evolve into exciting new directions (Lum, 2014). Media ecology probes the effects of anything we use in dealing with the world around us. For instance, McLuhan even includes lightbulbs as media. He does not focus solely on mass communication, but on how media enable us to do things. By his definition, media are extensions of the human body. They span bodily functions ranging from basic needs to cognition. This explains why McLuhan’s concept of media as “extensions of man” includes housing and cities as extensions of bodily heat control (McLuhan, 1964). This is obviously in addition to more classical areas he touches upon such as TV and movies (which extend our sensorimotor grasp) as well as the now-ubiquitous electronic media that are seen as an extension of the human nervous system (McLuhan, 1964, 1988).

Three things are relevant here. First, one of the more established distinctions in the amorphous field of media theory is its relative separation from communication theory. The latter predominantly focuses on the sender and the receiver, the source, and the destination of messages. Where communication theory describes what part of the message gets through (treating disturbances in the media channel as noise), media theory aims more directly at the media qualities of the given channel and the way external devices record, process, and convey information. Versions of communication theory based on Shannon and Weaver’s (1949) information model already had their impact on philosophy, such as in terms of the naturalization of intentionality in representational theories of mind (Dretske, 1981; Adams, 2003). It stands to reason that media theory could play a similar role within 4E cognition. Understanding the mind requires more than a focus on what information gets *in*. This understanding has to explain how mental states are brought forth in embodied engagements that are based on the cognitive practices I have described as joint explorations of media and organisms.

Second, media theory provides a way to centrally understand *culture* that spans technology and images, social engagement and art (Bickenbach, 2011). At the same time, it captures the decisive impact media have on the mind and the human sensorium (Gane and Sale, 2007; Jones, 2010). In this, it complements reconstructive evolutionary accounts of culture as social learning in biology (Heyes, 2020) and cognitive neuroscience (Gendron et al., 2020) by focusing on the aspects of learning and adaptation that are mediated by media. The humanities background for media theory could supply additional help in tracking the concept of value or significance across different disciplines, while also challenging conventional ways of thinking in the cognitive sciences. As an enactive category, artifactual habits involve more than just habituation. They decisively encompass a capacity to generate, sustain, and track values in the environment. Enculturation can then be understood as an extension of such value systems: “culture thus concerns all forms of significance that are common to groups of people and inherited by social rather than genetic means” (Durt et al., 2017, p. 74). 4E-supported media studies could explore enculturation by not focusing solely on social interactions with others (Veissière et al., 2019): it could instead achieve this by foregrounding the cultural artifacts and media domains that centrally permeate and structure our minds.

A third point, frequently made in media studies, is the claim the impact of media is so pervasive and ubiquitous, their co-constitutional role for our (cognitive) lives does not come to the fore anymore. As Bourdieu (1977) developed with respect to the concept of *doxa* (as opposed to the more explicit *dogmas* and norms in a society), culture could be seen as all the things that are taken for granted in a society. A theoretic effort is required to make explicit the ways in which we are enculturated. The reign media have over us is one that relates to their structural impact. This is captured in McLuhan’s most famous phrase: “the medium is the message” (McLuhan, 1964). In the sense of information or content, no message can measure up to the effects of the structural interaction enabled by the medium that carries the content. This makes McLuhan’s observation a theoretical call to the arms—one that extends to philosophy of mind that might be prone to miss out on the potentially profound impacts of media. It is therefore important to include a wide range of media and cultural artifacts to understand this impact (as I do in the next sections). While their impacts may not always be immediately transparent, they nonetheless form an infrastructural basis for experience and understanding.

## Toward a New Cognitive Media Theory

We saw that within a situated cognition perspective, some tenets of a general media theory could also constitute tenets for a philosophy of mind. Despite case studies in specific domains such as the internet (Halpin et al., 2010; Smart et al., 2017; Clowes, 2019), attempts to include a more general media theory within situated cognition are sparse.^19^ In media theory, *cognitive media theory* is most directly related to questions regarding the kind of mental states we entertain in our media engagements. These range from story engagements to aesthetic evaluations (Nannicelli and Taberham, 2014). For the remainder of this paper, I explore some media domains under its auspices. With this exploration, I intend to put the proposed artifactual habits account to work.

### The Filmic Body Schema

In the 1980s, film studies took a naturalistic turn that challenged the prevailing Big Theories of its time. The turn drew more systematically on research from linguistics, anthropology, evolutionary biology, psychology and neuroscience (Bordwell, 2013). The so-called ‘cognitive media theory’ claimed that the widespread impact of cinema “must be connected to some fairly generic features of human organisms to account for their power across class, cultural, and educational boundaries. The structures of perception and cognition are primary examples of fairly generic features of humans” (Carroll, 1985, p. 92). Filmmakers achieve their effects by eliciting emotions and guiding our attention by story and character development—but also by framing, camerawork, and editing. In this respect, movies are *attentional engines* (Carroll and Seeley, 2013; Seeley, 2020). Cognitive film theory never explicitly stated that it is committed to a basic set of cognitive mechanisms. It nonetheless rests on a fixed-properties view of the mind that the present paper wants to challenge by providing a more integrative and dynamic theory regarding our cognitive capacities.

An often-reported finding is the amount of viewer synchrony during feature films. Through an inter-subject correlation analysis of fMRI data from participants watching a movie (*The Good, the Bad, and the Ugly*), Hasson et al. (2008) found an exceedingly high convergence of activity. As other studies have confirmed, such convergence is higher for edited film clips compared to unedited ones (Herbec et al., 2015). This could support the universalist claim of cognitive media theory because it appears to establish the existence of generic features of the human cognitive system that cinema plays to. Edited sequences entrain us in their unfolding more than non-edited ones, as do moving images more so than static pictures. The latter claim been demonstrated with respect to “attentional synchrony” using eye-tracking paradigms: compared to static scenes, sequences with actions and movement generate greater attentional synchrony, with respect to fixations and saccades in participants—especially when tracking people and faces (Smith and Mital, 2013).

The general attentional synchrony for dynamic scenes has indeed been exploited by film to hide its media features (e.g., camera movements or editing). Particularly for Hollywood cinema, montage adheres to what has been labeled ‘continuity editing’; these are shooting and editing rules aimed at creating smooth, visual continuity in the eye of the beholder (Berliner and Cohen, 2011). The rules include perspectives and camera angles that can be assembled together before and after a cut (for instance, one should remain within an angle of 180 degrees and not go below 30 degrees). Often, the movement of an object or person is preserved when there is a cut. This ensures that such “match-action” cuts keep us entrained (Smith and Martin-Portugues Santacreu, 2017). By employing these techniques, there is a high propensity that our engagement with medium-specific characteristics such as edits does not reach conscious awareness anymore (Fingerhut, 2020b), or, at the very least, are subdued. This is captured by a phenomenon called ‘edit blindness.’ 30 percent or more of cuts go unrecognized within a scene, even when the viewer is tasked solely with reporting cuts in 5-min clips from Hollywood blockbusters (Smith and Henderson, 2008).

From the perceptual cognitive neuroscience perspective, each cut constitutes a significant event or violation of expectations. The neural signature of a film cut resembles that of a syntactic violation in language processing or in the order of sequence for comic-like stories using static images (Magliano and Zacks, 2011; Maffongelli et al., 2015). Let’s return to the cuts described above. When comparing continuity edits to those that depart from the rules, no significant differences in early visual or syntactic processing were found. Instead, differences appear in brain areas that process violation repair. In cases where such post-perceptual updating is not occurring, other areas (such as those related to the conscious processing in detection tasks) are found to have neural signatures resembling those when a change is detected in a change blindness paradigm (Heimann et al., 2017).

One interpretation of these findings is that the visual entrainment to depicted elements (perhaps the movement of an object or person before and after a continuity edit) is sufficient to suppress conscious processing of cuts, allowing viewers to engage with the scene. In non-continuity editing, those content-related cues are simply insufficient to suppress awareness of the filmic means.

Yet one could also argue that continuity editing only works because it is integrated into a learned habit of enacting film. This would mean editing recedes into the background (and escapes our attention) only after we have developed a pictorial, moving-image competence. First, we must have had some exposure to edited film. Only once we have incorporated our filmic explorations (through camera and editing) into an artifactual habit of perceiving, may we stop perceiving these discrete configurational elements as independent elements, or events. Indeed, there is some experimental support for such a view. First-time viewers of film do have trouble perceiving spatiotemporal continuity in a scene that is put together adhering classic editing rules. Due to cuts and perspectival changes, such viewers do not perceive what is depicted before and after the cut as one and the same object (Ildirar and Schwan, 2015; Ildirar and Ewing, 2018). One explanation for this is that first-time viewers perceive cuts as a strong distortion—not just as a perspectival shift displaying the same scene. The flipside of this is experienced viewers of film have integrated such violations as part of film viewing and have developed a filmic habit of engagement. This then can be seen as one element of a filmic habit that comes with its own sensorimotor rules or even body schema (Fingerhut and Heimann, 2017). And it is only *within* such a filmic body schema that we can explain how attention and emotions are employed while experiencing a story in a way that captures what makes our engagement special in such cases.^20^

The present paper assembles phenomena from different media domains, thereby exploring how best our cognitive engagement may be described. This includes focusing on how external media and neural processing should be combined in terms of the realization base of mental states as well as focusing on enactive sense-making and the habits that structure such sense-making in different media ecologies (with habits constituting a central level of description in 4E media theory). I furthermore argue that predictive theories could fit neatly into this picture, for they can explain how we engage with media works (e.g., what predictions we bring to bear when we, for instance, watch a melodrama, a TV crime series, a horror movie, or read a novel). The more radical version of predictive processing discussed may additionally capture how we share into the explorative world-models designer environments present us, rendering them mutual models.

I am not aware on any substantive work on predictive coding and film. Nevertheless, there are interesting attempts to apply predictive models to works of literature, namely by treating literary texts as *probability designs* (Kukkonen, 2014, 2020). Generally, Kukkonen argues that literature engages in enhanced interoceptive explorations, referring to claims that inferences in hierarchical PC encompass exteroceptive and interoceptive prediction errors alike (Seth, 2013). Since the medium (in this instance, texts) limits our range of actions within a media environment, it makes specific elements more salient, allowing us to further explore affective evaluations of our inner realms that might otherwise go unrealized. Predictions here unfold on several levels, the most important one addressing narrative and plot. Given my focus on sensorimotor and body-schematic processes, I am more interested in the embodied reading experience and the designed sensory flow in such engagements. Here, *form* emerges as the central concept. Form, which is “foregrounded in the designed sensory flow of the sentences[,] sparks epistemic active inference, but arguably [it] also [serves] as [an anchor] in the text to return to” (Kukkonen, 2020, p. 189). Because real-world bodily engagement is attenuated in reading, literary structures and formal elements can channel sensory flow in media-specific ways. On this point, compare how, in film, both editing and camera work scaffold our immersion and determine our engagement. However, the ability to return to those specific anchors earlier in the film experience is largely precluded. Therefore our self-initiated embodied engagement might be even more reduced compared to literature (in which we could, e.g., saccade or scroll back to earlier passages in the text). In engaging a filmic body schema under cinematic conditions we surrender our motor activity to the medium. This therefore constitutes a different trade-off between extero- and interoception by contrast with literature.

### Seeing-In Pictures

The discussion of body schemas and pictures can also be couched in a broader question: what is the main difference with respect to the skills and habits that we bring to bear in pictorial perception compared to those we employ in the real world? Let’s consider, for a moment, static images such as drawings, paintings, and photos. Such pictures are peculiar kinds of objects. I have argued elsewhere (Fingerhut, 2014, 2020a; Fingerhut and Heimann, 2017) that pictures (i) afford specific epistemic operations, that they are (ii) affective objects that can address us in powerful ways, and (iii) that via exposure and experience-based learning, we develop an artifact-specific perceptual manner of engagement with them. The latter aligns mostly with the topic of the present paper. To properly address our pictorial habits of perceiving, consider again the insight from enactive sense-making: cognizers must actively bring forth experiences. Enacting what we experience takes a different turn when we engage with pictures. The reason for this becomes obvious when we think about the sensorimotor patterns involved. Changing our position relative to the picture, for instance, does not allow us to see behind a depicted object. Pictures and depicted objects thus provide their own—and sometimes paradoxical—experiences of presence (Noë, 2012; Seth, 2019). Material pictures afford a different kind of exploration with respect to what is depicted (their content) and with respect to the properties of their surfaces (their configurational features). But most crucially, we experience a *surface-content relation* when we see a picture. In fact, it has been argued that perceiving pictures is constituted by engaging such a surface-content interaction; it relies on the cognitive operation of *seeing-in* that comes with the phenomenology of a *twofold* experience (Wollheim, 1980/2015; Hopkins, 2003; Lopes, 2003). To perceive something in a picture, we have to engage with its configurational and with its representational properties. Both jointly constitute the experience.

The intricacies of the philosophical debate regarding seeing-in are not relevant for the present paper as the point I would like to make is more general. It seems obvious here that perception of the surfaces of pictures and perception of what is depicted afford different sensorimotor operations. Yet it is the *interaction, parallel processing*, or *integration* of the two operations within the habit of picture perception, in particular, that must be better understood. This, strangely enough, is largely ignored in the cognitive sciences that use pictures as stimuli and even the field of neuroaesthetics (Fingerhut, 2018b).

Consider embodied simulation accounts that highlight motor responses as a necessary feature of our engagement with pictures such as paintings (Freedberg and Gallese, 2007). They focus on body postures, implied actions, and the facial expressions of depicted human figures on the one hand, and on premotor areas responding to perceived brushstrokes or cuts of the canvas on the other (Umilta’ et al., 2012; Sbriscia-Fioretti et al., 2013). Nonetheless, they do not explore how both folds of our cognitive processing (i.e., of surface and content) interact in our engagement with a painting. That is, they do not show how the parallel motor processing of surface and content features determine our experiences in such cases.^21^ I do not believe this is a minor point: if our perceptual habit of picture perception is defined by this double processing, then this is a necessary complication for any theory of pictorial engagement (Fingerhut, 2018a). This last point more generally attests to the need to study habits as a unit rather than as something constituted by disjointed processes. In order to understand picture perception, the intertwined processing of configuration and content afforded by those artifacts has to be taken into account.

It has been argued that film does not have a surface in the same way other pictures have and that therefore there is no seeing-in with respect to film (Cavell, 1979; Carroll, 1996). This can be illustrated by the central role of sensorimotor engagement with the surface of a handmade painting: moving toward a painting makes the brushstrokes more visible and might contribute to the central experience of the artwork (Currie, 2018). This does not occur in the same way with the surface on which film is shown, such as a projection screen in cinema. Nonetheless, there is good reason to extend the notion of seeing-in to moving images and the many screen-based digital media containing them. Also in film we interact with configurational features (edits, camera, and lens movements) and the evolving content simultaneously. As with representational static images, any account of our filmic habits would have to integrate this double engagement and explain how film actively guides our exploration through specific moving-image strategies (Fingerhut, 2020b).

Such a focus could constitute one way to complement the more generic features of our cognitive apparatus described in cognitive media theory. Yet it should come as no surprise that also other expansions of cognitive engagements through the medium of film have been explored in the literature. One example is empathy. It has been argued that film affords expansive empathic engagement by providing close-ups that, for instance, enable us to engage more intensely with the faces of depicted characters. This engagement facilitates a better understanding of people from what could be considered outgroups and to which we otherwise would not develop such an involvement. Smith (2012),BR171 discusses this within a 4E framework by referring to the aforementioned embodied simulation accounts (motor simulation of facial mimicry and observed actions). Embodied simulation functions as a mediator to enhance our engagement with characters that we would not have the same access to under normal conditions.

The kind of motor activity described by Smith is seen as having the domain-general function of facilitating empathy. Film thus expands some of the features (through close-ups of faces, gestures, etc.) we can pick up on as well as the class of organisms or objects (marginalized groups, aliens, robots, villains, etc.) to which we allot this kind of empathy. This is important in of itself. But what I want to add is that Smith’s application of motor theories of empathy still relies on a bio- or socio-chauvinistic interpretation of neural activity. As I have argued above, such a view needs to be amended by a focus on the *neuromedial* elements that are part of the larger, structural way a movie recruits and engages the cognitive apparatus within our filmic habit. Motor activity is also modulated by filmic features such as camera movements and edits (Heimann et al., 2014, 2019) and therefore configurational features of the medium. We have to take into account how these have been incorporated into our ways of exploring a scene in film. This is what a *new cognitive media theory* should capitalize on. So in terms of the motor-empathy framework discussed in the preceding paragraphs, one could speak of “empathy with the medium”—one that not only includes the depicted persons *or* the stylistic means of film independent of each other, but centrally the integrated seeing-in habits related to moving images (i.e., the interplay of configuration and recognition in our engagement of film, see Fingerhut, 2020b). Any neural activity, and especially the neuromedial side of the larger artifactual habit, would have to be interpreted with respect to such normal conditions of film perception.

### Digital and New Media

Pictures and moving images are intimately woven into recent digital revolutions. Concepts such as post-cinema or trans- and intermediality in storytelling capture only some of ways that images migrate or are processed therein. The presence of screen-based media is permanent both as portable devices and stable within our environment. Data from our interactions with such interfaces are fed back into what is presented on them (think of data-mining artificial intelligence in social media). The term ‘new media’ largely designates the field of social media, sometimes including the devices and gadgets used to engage with this particular media. But it also marks something that is akin to all media and fits the third notion of *expansiveness* from above: “by changing the conditions for the production of experience, new media destabilize existing patterns of biological, psychical, and collective life even as they furnish new facilities” (Hansen, 2010, p. 173). In this sense, old new media (the emergence of cave paintings, the printing press) might already reveal many things that can be applied to more recent new media as well (Manchovic, 2001), and could also help us understand our intensely digitally mediated environments.

In the expansive habits view I have proposed, new and digital media are interesting for many reasons. Such media create enhanced dynamics due to parallel available and transmedia ecologies that require an additional focus on the meta-habit of switching between multiple platforms, formats, and devices. However, I will focus on two central points only. First, digital media are not disembodied media. Their interfacing devices exploit existing embodied engagements by aiming to be more seamlessly integrated than other media have been to date. Second, media devices evolve in rapid reciprocal adjustments with users. Now, there are even media set-ups that employ real-time feedback loops and real-time adjustment to the organism. This relates to the growing domain of pervasive and ubiquitous computing in the background of our world (Lyytinen and Yoo, 2002) and to the algorithms and artificial intelligence (AI) used to predict our interests (as evidenced by various functions on Facebook, Twitter, Snapchat, TikTok, and so on). Such predictive activity emanating from the backend of media corresponds with the concept of *neuromediality* in an interesting way. Now, this concept denotes the neural contribution within a habit not only on the side of the organism but is also employed within the external medium itself. Today, media environments themselves operate under neural regimes.

Coming back to the first point I want to highlight. This involves embodied routines of interaction and the ways our bodily gestures (as well as those related to older media artifacts) became integrated into novel interfaces. Think of our use of touchscreens via gestures. A small but important point in this respect is that even such seemingly seamless devices do nonetheless require specific media skills (and related sensorimotor and body-schematic processes).

This has been demonstrated by developmental psychology and research into the so-called ‘video deficit effect,’ or the ability to transfer learned content from 2D to 3D to real-life-situations. Such transfer ability is relatively poor in infants (Anderson and Pempek, 2005). This means that media skills cannot be immediately applied in a domain-general way and as easily be transferred between media and outside the media context.

Recently, this kind of research has been extended to study what it means to grow up in new digital environments (Barr, 2019). Touchscreen devices appear to provide more interactive opportunities that should make transfer to 3D worlds outside the media context more immediate. Yet transfer deficits nonetheless remain also for touch screens. For example, children who learn to press buttons on a 2D touchscreen cannot use this skill with respect to 3D objects as immediately as one might expect (Zack et al., 2009). The overall point of such findings is that despite a general ability to transfer recognition and action skills between media, or between media and the real world, such transfers often come at a cost, such as additional cognitive load (Zack et al., 2013). While such a load seems to be neglectable and often remains unnoticed in adults, studies with infants provide some support for the claim that media habits require their own rules of engagement, even in media that seem to have adapted to the human motor-sensorium.

The second aforementioned aspect refers to the content and configuration of new digital media being adjusted in ever-shorter timescales (up to real time) to their users. A common example is learning software that adapts to the skillset of its user. Likewise, our choices determine the content portrayed to us in social media. Such responsive feedback is also at the heart of the concept of *enactive media* (Tikka, 2010a; Kaipainen et al., 2011). The structurally interesting features of such media is that they pick up on our actions and physiology and adjust their feedback accordingly. The authors describe one specific filmic media setting in which “technology is a part of a two-way feedback system with self-controlling recursive properties, and the role of an interface becomes implicit, perhaps even to the degree of being non-conscious” (Kaipainen et al., 2011, p. 433). The relevant cinema installation includes a montage machine unit that recombines elements from a database into cinematic composition based on psycho-physical data from the viewer (see also Tikka, 2010b). This makes the viewer the unconscious author of their media content.

Despite the focus on cinematic narrative, the discussion of enactive media has a more general relevance. For one, it makes explicit the possibility of new media systems to attune their user in real-time by in future also more systematically mining their physiological and neural data. For another, it simultaneously limits cognitive access to the interface of such adjustments. Much more could be said about whether enactive media introduce a new dynamicism from the artifact side, or whether they simply demonstrate more clearly how media always have entrained and transformed us. I included them in the present paper to demonstrate that a *new cognitive media theory* must not simply highlight media-specific abilities (artifactual habits beyond the generic cognitive abilities addressed by cognitive media theory). It must also address the dynamic reciprocal influences of organism and media environments, which both enactivism and RPP have made salient. Such dynamics might include a highly adaptive (and thus *neuromedially predictive*) element on the media artifact side as well. This element could change the character of media-related habits that already encompass artifact and organism (a I aimed to capture by the concept of *locational expansiveness*). Such neuromedial elements on the artifact side renders organism and media artifacts ever more intimately interwoven.

### Architecture and Cities

Media have been treated as extensions of our bodies. McLuhan’s media cases thus include buildings and cities, which are viewed as extensions of our metabolic system. But the built environment structures cognition and actions on a multitude of levels; it affects us continuously across all of our senses from vision to the vestibular, from touch to sound. We create our reality as we move through designed space. The impact of architecture and design remains a largely understudied field in philosophy and cognitive science. This is certainly true compared to study of language, but also compared to study of pictures and even computation and digitalization. Still, things have started to shift due in part to scholarly interest in the possible convergence of embodied cognition paradigms and architectural studies (Mallgrave, 2013; Pallasmaa et al., 2015; Robinson and Pallasmaa, 2017).

Currently, half of the world’s population lives in densely populated urban areas. This portion is projected to rise above two thirds of the population by 2050. Recent studies have explored correlations between cities and mental health, noting that the risks for anxiety disorders and psychotic disorders such as schizophrenia might be significantly higher in cities (Gruebner et al., 2017; but see DeVylder et al., 2018). It thus seems pressing to study the impact of architecture and city planning, along with general urban cognitive ecosystems, on mental well-being, cognition, and experience. Some emerging fields, such as *neurourbanism*, do so (Adli et al., 2017; Fett et al., 2019). Essentially, the built environment is the ultimate designer environment for our embodied minds to fold into their cognizing and experiencing. This is because it is such a determining factor across a wide range of bodily actions.

It is worth briefly considering the constant and stabilizing influence of the built environment on our habits of engagement. Due to its continuous presence, we might overlook its impact. This would render architecture-related perceptual engagements a human constant that is no longer a visibly part of an artifactual habit. Still, there are some indications of how the built environment might have permeated our perception. One example is the Müller-Lyer illusion (which portrays two lines of equal length as different lengths to the human vision, thanks to fins at the end of the line protruding either outwards or inwards). The illusion appears to be universal. For instance, it is present in children who gain sight after congenital blindness (Gandhi et al., 2015). Yet the size of the effect is not universal. It has been smaller for Navajo native Americans who grew up in traditional roundhouses compared to those who grew up in new reservation architecture (Pedersen and Wheeler, 1983; Phillips, 2019). This has been related to a the ‘carpentered world hypothesis’. The rationale is that we perceive lines with fins protruding outwards as being at the back of a room (or of something else in our carpentered worlds). They appear enlarged in our perception because the visual system compensates for them being seemingly further away.

Other studies have focused on the impact of navigation in cities on our cognitive system. In a seminal study on experience-driven neuroplasticity, taxi drivers in London showed greater gray matter volume in the mid-posterior hippocampi compared to bus drivers who do not have to exhibit the same navigational skills (Maguire et al., 2006). A more general exploration of the navigational capacities of 442,195 participants across 38 countries by the same lab found participants raised in cities had worse navigation skills than those raised in more rural areas. The effect was larger for cities that had a geometric grid layout compared to more organic and complex ones (Coutrot et al., 2020). The taxi driver data reflects a task-driven plasticity, while the city-rural comparison shows more generally how an environment recruits its organisms and then alters their cognitive capacities. The data therefore indicate that statistical immersion to an environment alters our embodied, cognitive habits and that organisms allocate neuronal processing resources (and undergo structural changes) according to the demands of their environments.

The co-dependency and reciprocal shaping of architectural and human embodiment also happens over smaller and dynamic timescales (Jelić et al., 2016). The stable presence of architectural elements has a corollary effect (in keeping with the effects of precision weighing in PP discussed above) wherein small changes have rather big impacts. A central architectural element are entrances and doors that afford locomotive permeability. They have been, for instance, explored in EEG experiments that measure motor preparation in the perception of such apertures, which showed a highly fine-tuned sensitivity to this particular architectural element (i.e., whether a door is walk-through-able or not, see Djebbara et al., 2019, 2021). Such adjustments are part of our architectural, multisensory habit to perceive architectural affordances. Within such sensorimotor engagements, we can understand how our experience of the built environment unfolds. Here, we pick up on a multitude of design decisions and architectural features in a dynamic way.^22^

After the initial interest from McLuhan (1964, 1988), buildings and cities did not become a central concern for media theory (but see Kittler and Griffin, 1996). As multisensory and mixed media environments, cities and architecture have re-entered the media theory landscape only recently (McQuire, 2008). Part of the reason for their return is the rise of ubiquitous and pervasive computing in smart cities. Artificial intelligence, the *Internet of Things*, and large-scale data analytics are now employed to predict and influence behavior. In this context, “architecture provides a fixed form for the flows engineered by pervasive computing” (McCullough, 2007, p. 395). Social media for city experiences (Molinillo et al., 2019) and sensory feedback loops in buildings might themselves become a central part of what we consider architecture in the future as they latch onto our already artifactual habits of engagement.

This section has described how artifactual habits relating to urban and architectural design entrain our perceptual engagement and determine cognitive capacities. The built environment presents us with experiential models in ways that are comparable to other media. Design decisions and urban planning provide different models for how we may live together. They influence urban dwellers in terms of their social behavior or explorations of their environment. By focusing on how design decision nudge us in cities and buildings (even without their ‘smart’ extensions), those cities could be described as media. They process, store, and transmit information, yet over longer timescales compared to other media. At the same time, they are projections of the kind of social being that a certain culture aims to produce and promote (for some critical implications of this, see Crippen and Klement, 2020). As such, architecture and the built environment are models of who we are (have been and will be) as a society.

## Conclusion and Outlook

Media environments and technologies evolve with our embodied brain-body nexus in reciprocal co-adaptations. In this, they constantly reconfigure and transform how we engage and experience. I have aimed to capture some of these dynamics by highlighting the expansive artifactual habits we entertain (because we live in a built environment, among a plethora of pictures, and now are immersed in new digital media that respond dynamically to us). I have mainly discussed media artifacts from pictorial domains at the omission of other elements or mixed media environments (such as sound and spoken language, texts, and how those are interwoven and interact with pictures) because I see images as underrepresented in the discussion of the relation of culture and mind. But I also aimed at a more general point: media in all their ramifications should occupy a central place within the still-maturing field of situated cognition.

I have therefore focused on a rather general concept in the philosophy of mind, namely habits (Caruana and Testa, 2020). With this, I sought to capture the basic insight into the relational nature of our mind propagated by 4E and enculturation theories alike: our mind is crucially determined by the embodied actions afforded by our socio-techno-cultural environments. As I introduced them, habits are critical qualifiers of the range of such actions within a specific ecology. In media ecologies, we are expert perceivers without knowing it. The way we explore the contents of different media is couched in habits that are partially constituted by the structural features of the media artifacts themselves. They are not rigid mechanical routines. Instead, habits are flexible ways of world-making.

I have only briefly tapped into the rich and evolving field of media studies by highlighting some general claims regarding media archeology and ecology. More specifically, I have addressed the way cognitive media theory captures our media engagement. Although this media theory has recently started to include ideas from situated cognition, I suggest that there are limitations to this account. In comparison, the pluralistic and dynamic view of artifactual habits (along with the interlocking of media and neuro-cognitive architecture) in my enactive account of media constitutes a larger shift in thinking. This shift might warrant the label of *new cognitive media theory*. Regardless, it entails acknowledgment of the plurality of habits and related bodily engagements (I discussed the filmic body schema we entertain when engaging with the pervasive artifact of moving images, as well as the capacity of seeing-in that pertains to all pictorial domains). It further offers an ensuing understanding of how our perceptual, emotional, and aesthetic engagement unfolds *within* such habits based on new insights into our cognitive apparatus.

No survey of situated or 4E accounts can be exhaustive. The field has evolved so rapidly that one is liable to miss out on developments even for subdomains like media engagement, which – unduly to my mind – are treated only at its periphery (I am, for instance, well-aware that I largely ignored phenomenological and post-phenomenological thinking regarding media). I aimed to capture some central junctures to the artifactual habits account of media I propose. Thus, I aimed to re-territorialize extended mind claims to sociocultural media-ecologies while retaining some of their focus on mixed-media coalitions *within* habits. I did not focus on the ontological claims related to this. Instead, I proposed an enactive understanding of how cultural artifacts have become integrated into our cognitive routines. As central element, they do so by bringing forth experiences in domains that sustain their own rules and values. I argued that radical predictive processing (RPP) could provide an accompanying explanation of how the nervous system facilitates organism-artifact coalitions and how we attune to design environments on multiple levels.

Our ability to engage with a plurality of designed media models captures something central and defining in human cognizing and experiencing. Once we understand the expansive artifactual habits that bring forth novel meanings and values, we can understand how our mind is mediated and becomes re-mediated at every moment of being engaged with such models. RPP served to situate the more local neuronal contribution within this larger picture; it elucidates a possible role of the brain in folding designed, media environments into our embodied engagements. Further, the concept of *neuromediality* captures some of this. It brings into focus the exapted functions certain neuronal processes might take on in different media ecologies. As such, neuromedial processes are part of the normal conditions of any media engagement. In recent digital media developments, neuromedial processes could even be ascribed to media themselves (as we saw with respect to the real-time dynamics of adapting and predicting their users).

This paper aimed to contribute to a broader understanding of enculturation in situated cognition by focusing on how we actively bring forth experiential models of the world that become salient through and within media. It did not address what could be considered our aesthetic relations to such cultural artifacts. Media and cultural artifacts actively invite our exploration of the world. They also invite evaluation of their ways of worldmaking. Aesthetic and emotional appreciation might be a central way to track the bundles of perceptual, cognitive, and other effects presented to us by cultural artifacts (I explore such relations elsewhere, see Fingerhut, 2018b; Fingerhut and Prinz, 2020, forthcoming). Aesthetic evaluations of specific media outputs relate to normative claims. This poses a threat to a more comprehensive convergence between the humanities element in media studies and naturalistic explanations in the 4E cognitive sciences (Nannicelli, 2019). Future research will have to address this. One promising way could be to explore what the present paper has established as the more general value-generating enactive view of habits and the affective dimension of the respective media models this entails.

## Data Availability Statement

The original contributions generated for this study are included in the article/supplementary material, further inquiries can be directed to the corresponding author.

## Author Contributions

The author confirms being the sole contributor of this work and has approved it for publication.

## Conflict of Interest

The author declares that the research was conducted in the absence of any commercial or financial relationships that could be construed as a potential conflict of interest.

## Footnotes

1 As such, media concepts have already helped to structure some central debates in analytical philosophy of mind and consciousness. These include discussion of the analog or digital content of mental states, Dennett’s rejection of any identifiable or special neural medium of consciousness, and his claim that consciousness is “fame in the brain” unbound from any specific medium (Dennett, 1993, 2001). Clearly, media theorizing can be fruitful for a heuristic of the mind. However, this paper is more immediately concerned with external media and the role they might play in constituting mental states.

2 Much has been said about the extent to which inner neural and outer media processes must have similar processing properties to warrant parity of treatment. For a recent take on this, see Wheeler (2019). For a critical view on whether this introduces a mark of the mental based on properties of inner processing, see Di Paolo (2009).

3 See Menary (2007). For the human organism, the benefits of cognitive integration is that it enables us to do things “we otherwise could not do and [in] the transformation of existing abilities, making us smarter and better at difficult and demanding cognitive tasks” (Menary, 2018, p. 197).

4 The dynamic exploitations of different media across brain-body-culture boundaries then unfold *within* such habitual engagements. This paper takes up the differences *between* media-habits as well as the rules of engagement *within* a habit (i.e., the specific unfolding of cognitive processes within a skill). I will not focus on what could be considered our meta-habits of (wittingly or unwittingly) choosing different media resources for engagement. Still, the latter has become a central focus in understanding media economies that compete for our attentional resources (Crogan and Kinsley, 2012).

5 The exploration of a scene in film, for example, relies on camera work and editing processes that have happened in the past. The perceiving subject is thus an active partaker in the succession of frames in the *here and now*, yet also a passive perceiver in terms of the many past operational decisions that they cannot influence. For discussion of pictorial artifacts in this respect, see Fingerhut (2014).

6 Most EM theorists assume local, neural realizers when it comes to *conscious* mental states (and argue only for the extended nature of non-occurrent mental states, such as beliefs), whereas the dynamic-reciprocal accounts just mentioned also prominently address the unfolding of conscious experiences. Such accounts also encompass cultural phenomena (such as architectural contexts) that fall under the purview of what I call media (Kirchhoff and Kiverstein, 2019, 2020).

7 By highlighting affordances for action within the environment, ecological psychology (Gibson, 1979) shares several tenets with enactive perception; the terminology of ‘affordances’ is thus used across theoretical boundaries. Ecological psychology comes with a set of further theoretical commitments that are not central to what I do in the present paper; I thus do not discuss overlaps and differences between ecological psychology and enactivism. For some recent discussions of this, see Ramstead et al. (2016), Crippen (2020), and Feiten (2020). See also section “Neuromediality and Media Affordances” below.

8 Although it could be argued that the *sensorimotor enactivism* of O’Regan and Noë (2001) does not centrally capture this reference to affective states and the autonomous body in need of coupling (Fingerhut, 2012).

9 In this sense I would argue that it remains explanatory necessary to identify specific structures in the brain-body-world nexus (i.e., in artifacts and human bodies) that jointly realize those loops, while at the same time retaining the possibility that cognition in the relational sense might have no location proper (see for an excellent critical discussion of this: Walter, 2014).

10 For an excellent overview on current pragmatist theorizing on habits, see Caruana and Testa (2020).

11 For sociocultural affordances in social relations, see Ramstead et al. (2016). For social affordances in digital media, see Fox and McEwan (2017). For affordances in architecture, see Jelić et al. (2016) and Djebbara et al. (2021).

12 Clark argues that the actively inferencing organism is not decoupled from the environment. It constantly updates its predictions or priors in a way that they no longer resemble classical mental representations anymore (Clark, 2015b). Others argue that an enactive account of predictive engagement (PE) should further do away with inferences and models in the theory. Instead, it should directly focus on the situation dynamics of the whole system along with concepts such as “adjustment, attunement, and accommodation” (Gallagher and Allen, 2018). I am greatly sympathetic to their version of predictive engagement, but do not see it in strong opposition to my understanding of the RPP account presented above. As part of my survey of 4E and related accounts, I have chosen to focus on RPP because it is more directly geared to an understanding of media as designer environments and could be seen as an extension of the extended mind views developed earlier. In contrast, Gallagher and Allen (2018) focus on the dynamics of social interaction.

13 The formal description of systems that engages with active inference (i.e., described within the boundaries of a Markov Blanket) could also include elements outside the living organism. In this sense, it would be an outer layer of a nested system (Kirchhoff et al., 2018). But without further explanation, such an outer layer would still seem to remain at the periphery of what constitutes cognitive engagement. What I try to argue is that we attune to external models at different levels of our hierarchical generative model on the organismic side.

14 But see Anderson and Chemero (2018).

15 Although, such subversion could be attributed to certain domains of our digital media environment. Consider the rise of casual puzzle games such as Candy Crush and Gardenscapes, which achieved 180 million downloads by 2018 (Katkoff, 2019). Such games present players with successive puzzles of ever-so-slightly increasing complexity.

16 For the concept of epistemic action see Kirsh and Maglio (1994) and the discussion in Clark and Chalmers (1998).

17 Habits evolve and find new expressions in the succession of media forms (reading, e.g., transitioned while its medium changed from handwritten texts, to printed books, to current tablets devices) or they emerge as new habits of engagement as in the case of more radical technical or artistic innovations (think, again, of moving image devices).

18 I do not aim to capture the multi-faceted field of media studies, the scope of which goes well beyond this paper. One reason is because many accounts in media studies combine cultural analysis and the philosophy of technology with normative claims. This includes reflections on the tyranny of digital media and computational thinking (Stiegler, 2019), the implications of the “neuro-image” in socio-political terms (Pisters, 2017), and the aforementioned critical assessment of the attention economy (Crogan and Kinsley, 2012).

19 An exception is Logan (2013).

20 While continuity editing therefore holds some interest to film studies, it also is too limited in its purview. Film scholars aim instead to understand how editing mediates the emotional and Gestalt perception within our filmic habit of engaging (Pearlman, 2017).

21 Embodied simulation accounts of pictures and pictorial artworks have been criticized for relying on inner representations as mediating such experiences and therefore not being properly embodied (Gallagher, 2011). I will not go into the details of this discussion here. But I believe that a more enactive understanding of the role of the motor system as involved in preparation for actions, as Gallagher suggests, might preserve some of the insights of the embodied simulation theory of the arts (Fingerhut, 2018b).

22 This can be illustrated by the impact of sound within the built environment. For example, sonic feedback from our own movements (manipulated to low vs. high pitch) can influence how large or heavy we experience our body to be (Tajadura-Jiménez et al., 2015). This demonstrates our capacity for multisensory, fine-tuned adjustment based on normal conditions within an architectural habit.
